# The Effects of the Combination of Buckwheat D-Fagomine and Fish Omega-3 Fatty Acids on Oxidative Stress and Related Risk Factors in Pre-Obese Rats

**DOI:** 10.3390/foods10020332

**Published:** 2021-02-04

**Authors:** Bernat Miralles-Pérez, Maria Rosa Nogués, Vanessa Sánchez-Martos, Núria Taltavull, Lucía Méndez, Isabel Medina, Sara Ramos-Romero, Josep L. Torres, Marta Romeu

**Affiliations:** 1 Functional Nutrition, Oxidation and Cardiovascular Disease Research Group (NFOC-SALUT), Pharmacology Unit, Department of Basic Medical Sciences, Universitat Rovira i Virgili, C/Sant Llorenç 21, 43201 Reus, Spain; bernat.miralles@urv.cat (B.M.-P.); vanessa.sanchez@urv.cat (V.S.-M.); nuria.taltavull@urv.cat (N.T.); marta.romeu@urv.cat (M.R.); 2 Institute of Marine Research (IIM-CSIC), C/Eduardo Cabello 6, 36208 Vigo, Spain; luciamendez@iim.csic.es (L.M.); medina@iim.csic.es (I.M.); 3 Institute of Advanced Chemistry of Catalonia (IQAC-CSIC), C/Jordi Girona 18-26, 08034 Barcelona, Spain; sara.ramos@iqac.csic.es (S.R.-R.); joseplluis.torres@iqac.csic.es (J.L.T.)

**Keywords:** D-fagomine, fish oil, dyslipidemia, transaminases, inflammation, oxidative stress

## Abstract

The combined supplementation of buckwheat D-fagomine (FG) and fish omega-3 polyunsaturated fatty acids (ω-3 PUFA) attenuates the development of insulin resistance in rats fed a high-fat (HF) diet. This study aimed to examine the effects of combined supplementation with FG and ω-3 PUFA on dyslipidemia, transaminases, interleukin-6, and oxidative stress. Forty-five male Sprague-Dawley rats were fed a standard diet, an HF diet, an HF diet supplemented with FG, an HF diet supplemented with ω-3 PUFA, or an HF diet supplemented with FG and ω-3 PUFA for 21 weeks. Triacylglycerol, cholesterol, aspartate aminotransferase, alanine aminotransferase, and interleukin-6 were measured. The assessment of oxidative stress included plasma antioxidant capacity, antioxidant enzyme activities, glutathione content, lipid peroxidation, and protein carbonylation. The combined supplementation with FG and ω-3 PUFA did not attenuate the slight accumulation of liver cholesterol induced by the HF diet but normalized the plasma alanine aminotransferase activity. Rats fed the HF diet supplemented with the combination showed a lower amount of plasma interleukin-6 than those fed a standard diet. The combination attenuated oxidative damage induced by the HF diet, decreased antioxidant enzyme activities, and enhanced glutathione status. The beneficial effects of the combination of FG and ω-3 PUFA on oxidative stress and related risk factors in pre-obese rats were mainly modulated by ω-3 PUFA.

## 1. Introduction

Obesity is characterized by the expansion of white adipose tissue content due to an increase in both the number and size of adipocytes [1]. Concretely, visceral obesity is a central component of metabolic syndrome, which also includes dyslipidemia, impaired glucose tolerance, and hypertension [2]. The past few decades have seen an alarming increase in the worldwide prevalence of obesity [3], which has been related to a Westernized dietary pattern that is rich in saturated animal fat and sucrose and high in overall energy amounts [4,5].

Long-term high-fat (HF) feeding promotes oxidative stress [6,7], which leads to a disturbance in redox signaling pathways and/or molecular damage to lipids and proteins [8]. Early oxidative stress in expanded adipose tissue plays a significant role in the onset of obesity-related metabolic disorders such as insulin resistance, type 2 diabetes, and non-alcoholic fatty liver disease in rodents and humans [6,7,9,10,11]. Concretely, the aberrant accumulation of liver fat is also linked to oxidative stress, inflammation, and dysfunction of mitochondria, promoting the development of steatohepatitis and increasing the risk of fibrosis, cirrhosis, and hepatocellular carcinoma [12].

Dietary supplements and functional foods may prevent tissue dysfunction and the development of metabolic disorders by decreasing oxidative stress and inflammation. Individual d-fagomine (FG) and omega-3 polyunsaturated fatty acid (ω-3 PUFA) supplements attenuate the onset of insulin resistance in rats fed an HF diet [13,14,15,16,17]. FG is an iminocyclitol mainly present in buckwheat-based products [18]. Supplementation with FG improves glucose tolerance and reduces low-grade chronic inflammation, partially modulated by intestinal glycosidase inhibitory action and beneficial modifications in the gut microbiota [13,17,19]. The ω-3 PUFA, specifically eicosapentaenoic acid (EPA, 20:5) and docosahexaenoic acid (DHA, 22:6), are present in large quantities in fish and seafood products. Supplementation with ω-3 PUFA in a balanced EPA/DHA 1:1 ratio improves systemic antioxidant status, decreases inflammatory parameters in tissues, and prevents plasma dyslipidemia and the accumulation of ceramides in the liver [14,16,20].

Combining bioactive nutrients targeting both oxidative stress and inflammatory pathways may be a better nutritional strategy to prevent the onset of obesity and related disorders than providing them individually [21,22]. We have recently shown that the combined supplementation of both FG and ω-3 PUFA attenuates the increase in visceral adipose tissue content and fasting glucose concentration, plasma hyperinsulinemia, plasma hyperleptinemia. as well as lobular inflammation in the liver induced by an HF diet [23]. Moreover, the combination of these two nutrients promotes the growth of beneficial populations of Lactobacilliales and Bifidobacteriales and the production of short-chain fatty acids in rats receiving the HF diet [23].

Although ω-3 PUFA has been found to exhibit antioxidant properties by enhancing the non-enzymatic antioxidant capacity and endogenous antioxidant defenses [24], the complementary or synergic effects of FG combined with ω-3 PUFA on these parameters have yet to be assessed.

This study therefore expands upon our previous work and aims to examine the effects of combined supplementation with FG and ω-3 PUFA on dyslipidemia, transaminases, interleukin-6, and oxidative stress in pre-obese rats.

## 2. Materials and Methods

### 2.1. Ethics Statement

All animal methods comply with the European Union guidelines for the care and handling of laboratory animals (EU Directive 2010/63/EU). The permission was obtained from the Bioethics subcommittee of the Spanish National Research Council and the regional Catalan authorities (reference number DAAM7921).

### 2.2. Animals and Experimental Design

We purchased 45 male Sprague-Dawley rats (324 ± 19 g body weight; 8–9 weeks old) (Hsd:SD, Envigo, Indianapolis, IN, USA). The rats were kept in an insulated room (three per Makrolon cage; 425 × 265 × 180 mm) at a controlled temperature (22 ± 2 °C), humidity (60%), and 12 h artificial light/dark cycle.

Prior to the dietary intervention, the rats were fed a standard diet (STD; Teklad Global 14% Protein Rodent Maintenance Diet [2.9 kcal/g], Envigo, Indianapolis, IN, USA). After two weeks of acclimatization, the rats were randomly allocated into five groups (nine per group) and fed one of the following diets for 21 weeks: (1) an STD diet (STD group), (2) an HF diet (TD.08811 45% kcal Fat Diet [4.7 kcal/g], Envigo, Indianapolis, IN, USA), (3) an HF diet supplemented with FG (FG group), (4) an HF diet supplemented with EPA/DHA in a balanced 1:1 ratio (ω-3 group), or (5) an HF diet supplemented with FG and EPA/DHA in a balanced 1:1 ratio (FG&ω-3 group). The rats had ad libitum access to food and water (Ribes, Barcelona, Spain) throughout the study. The experimental diets are described in the Supplementary Material (Table S1).

The FG (>98%) was provided by Taihua Shouyue (HK) International Co. Ltd (Hong Kong, China) and was manufactured by Bioglane SLNE (Barcelona, Spain). It was included in the feed of the FG&ω-3 and FG groups at a proportion of 0.96 g/kg feed, as previously defined [19].

The EPA/DHA mixture in a balanced 1:1 ratio was obtained by combining the appropriate quantities of the commercial fish oils AFAMPES 121 EPA (AFAMSA, Vigo, Spain) and Omega-3 RX (EnerZona, Milan, Italy). Fish oil was administered once a week by oral gavage in the FG&ω-3 and ω-3 groups using a gastric probe at a dose of 0.8 mL oil/kg body weight. Soybean oil (Clearspring Ltd., London, UK) was administered by oral gavage in the STD, HF, and FG groups at the same time and at the same dose to compensate for the stress of probing and the excess of calories from fish oil in the FG&ω-3 and ω-3 groups. The fatty acid composition of the oils used is described in the Supplementary Material (Table S2).

Feed intake (g) was monitored daily. Subsequently, energy intake (kcal) was estimated using the Atwater conversion factors: 4 kcal/g protein, 9 kcal/g fat, and 4 kcal/g available carbohydrate.

Body weight (g) was monitored daily. Body weight gain (g), adiposity index (%, perigonadal adipose tissue weight (g)/body weight (g) × 100), and hepatosomatic index (%, liver weight (g)/body weight (g) × 100) were calculated at the end of the study.

### 2.3. Measurement of Glucose Tolerance

At week 18 of the study, an oral glucose tolerance test (OGTT) was performed on fasting animals. Before the test, glucose was administered by oral gavage (1 g/kg body weight), and blood glucose concentration was measured 15, 30, 45, 60, 90 and 120 min after glucose intake. Blood glucose was measured by means of the enzyme electrode method, using an Ascensia ELITE XL blood glucometer (Bayer Consumer Care, Basel, Switzerland). The results were expressed as area under the curve (AUC, mg/mL per 120 min), calculated using the Trapezium method.

### 2.4. Sample Processing

After 21 weeks of dietary intervention, the rats were fasted overnight, anesthetized intraperitoneally with ketamine (80 mg/kg body weight) and xylazine (10 mg/kg body weight), and sacrificed by exsanguination. Blood samples were taken by cardiac puncture. Then, plasma was obtained by centrifugation at 850× *g* for 15 min at 4 °C. After the removal of plasma, erythrocytes were obtained by washing twice with 154 mM of sodium chloride solution and centrifugation at 1300× *g* for 5 min at 4 °C. Plasma and erythrocyte samples were aliquoted and stored at −80 °C until use.

Samples of perigonadal white adipose tissue as a biomarker of visceral adiposity and liver were collected, washed with 154 mM sodium chloride solution, weighed, and cut. After that, adipose tissue and liver samples were snap-frozen in liquid nitrogen and stored at −80 °C until use. Frozen adipose tissue samples were homogenized on ice in 200 mM of sodium phosphate buffer (pH 6.25), sonicated for 1 min, and centrifuged at 1000× *g* for 10 min at 4 °C. The soluble fraction was then carefully collected and centrifuged at 129,000× *g* for 1 h at 4 °C. Frozen liver samples were divided into two parts. One part of the liver sample was homogenized on ice in 154 mM of sodium chloride solution containing 0.1% Triton X-100 (*v*/*v*) and centrifuged at 3000× *g* for 5 min at room temperature for the measurement of lipid content. The other part was homogenized on ice in 200 mM of sodium phosphate buffer (pH 6.25) and centrifuged at 129,000× *g* for 1 h at 4 °C for the measurement of oxidative stress biomarkers. Tissue samples were aliquoted and stored at −80 °C until use.

### 2.5. Measurements of Insulin Resistance Biomarkers

At the end of the study, the fasting blood glucose concentration was measured as described above for the OGTT. Fasting plasma insulin concentration was measured using the corresponding ELISA kit (Millipore Corporation, Billerica, MA, USA).

### 2.6. Measurements of Lipid Profile, Transaminases and Interleukin-6

Plasma triacylglycerol (TG), total cholesterol (TC), high-density lipoprotein cholesterol (HDL), and low-density lipoprotein cholesterol (LDL) concentrations were measured by means of colorimetric enzymatic methods using the corresponding commercial kits (Spinreact, Girona, Spain) in a COBAS MIRA autoanalyzer (Roche Diagnostics System, Madrid, Spain). In addition, the LDL/HDL ratio was calculated. Liver TG and TC contents were measured as described above for plasma.

Plasma aspartate aminotransferase (AST) and alanine aminotransferase (ALT) activities were measured by means of spectrophotometry using the corresponding commercial kits (Spinreact, Girona, Spain) in a COBAS MIRA autoanalyzer (Roche Diagnostics System, Madrid, Spain). In addition, the AST/ALT ratio was calculated as a biomarker of liver function.

Plasma interleukin-6 (IL-6) concentration was measured using magnetic bead Milliplex xMAP multiplex technology with the corresponding ELISA kit (Millipore Corporation, Billerica, MA, USA).

### 2.7. Measurements of Oxidative Stress Biomarkers

#### 2.7.1. Plasma Non-Enzymatic Antioxidant Capacity

Plasma antioxidant capacity was assessed using the oxygen radical absorbance capacity (ORAC) [25] and the ferric reducing ability of plasma (FRAP) assays [26]. The ORAC was measured using a Fluoroskan Ascent microplate fluorimeter (Labsystems, Helsinki, Finland), and trolox as a standard (Sigma-Aldrich, Madrid, Spain). The FRAP was measured using a PowerWave XS2 microplate spectrophotometer (Biotek Instruments Inc., Winooski, VT, USA), and trolox as a standard.

#### 2.7.2. Antioxidant Enzymes and Glutathione

Superoxide dismutase (SOD), catalase (CAT), glutathione peroxidase (GPx), and glutathione reductase (GR) were assessed in erythrocytes, adipose tissue, and liver. The total SOD and CAT activities were measured according to the methods developed by Mirsa and Fridovich [27] and Cohen et al. [28], respectively, using a Lambda 25 UV-Vis spectrophotometer (Perkin Elmer, Shelton, CT, USA). GPx and GR activities were measured according to the method developed by Wheeler et al. [29] using a COBAS MIRA autoanalyzer (Roche Diagnostics System, Madrid, Spain).

Reduced glutathione (GSH) and oxidized glutathione (GSSG) in plasma, erythrocytes, adipose tissue, and liver were measured according to the method developed by Hissin and Hilf [30] using an LS55 fluorescence spectrophotometer (Perkin Elmer, Shelton, CT, USA) and the corresponding standard curves (Sigma-Aldrich, Madrid, Spain). In addition, the GSSG/GSH ratio was calculated as a biomarker of the redox state.

Measurements in erythrocyte samples were normalized to hemoglobin (Hb) concentration in total blood. Hb concentration was measured according to the Drabkin method [31] using a Lambda 25 UV-Vis spectrophotometer (Perkin Elmer, Shelton, CT, USA), and an Hb standard (Spinreact, Girona, Spain).

#### 2.7.3. Lipid Peroxidation and Protein Carbonylation

Lipid peroxidation (conjugated dienes hydroperoxides) and total protein carbonylation in plasma and liver were measured as previously described [32]. Briefly, total lipids were extracted and quantified. After that, conjugated dienes hydroperoxides were measured in a Beckman DU-640 spectrophotometer (Beckman Instruments Inc., Palo Alto, CA, USA). Proteins were extracted and the protein carbonyl content was labeled with fluorescein-5-thiosemicarbazide and detected by one-dimensional sodium dodecyl sulfate-polyacrylamide gel electrophoresis.

### 2.8. Statistical Analysis

The statistical analysis was performed using IBM SPSS v.25 software (IBM, Chicago, IL, USA). Samples were assayed in duplicate. The results are expressed as mean and standard deviation and the Shapiro-Wilk test was used to test for the normality of data. Groups were then compared by means of the one-way analysis of variance, followed by Scheffé post-hoc test or the non-parametric Kruskal-Wallis test followed by the Mann–Whitney U test. The level of statistical significance was set at a *p*-value < 0.05.

## 3. Results

### 3.1. Feed Intake, Biometric Data and Insulin Resistance Biomarkers

Because these parameters have been partially described and discussed in previous work [23], we include these data here as Supplementary Material (Figure S1 and Table S3). All HF diet-fed groups presented a lower feed intake and higher energy intake than the STD group throughout the study. Although no significant differences were found in either final body weight or body weight gain among the groups, the HF diet increased the perigonadal white adipose tissue content after 21 weeks. Even so, both groups supplemented with FG, either individually or in combination with ω-3 PUFA, showed similar adipose tissue content to rats fed an STD diet. In contrast, the individual ω-3 PUFA supplementation did not attenuate the increase in adipose tissue content under HF-feeding conditions. No significant differences were found in liver weight among the groups.

At week 18, all the groups showed similar glucose tolerance in vivo. However, the HF group registered higher fasting blood glucose and fasting plasma insulin concentrations than the STD group by the end of the study. All the supplementations attenuated the increase in insulin concentration under HF-feeding. Nevertheless, the increase in fasting blood glucose was only attenuated by the combination of FG with ω-3 PUFA.

### 3.2. Lipid Profile, Transaminases and Interleukin-6

Both groups supplemented with ω-3 PUFA, either individually or in combination with FG, as well as the HF group registered lower plasma TC concentrations than the STD group, mainly due to the decreased HDL concentrations in these groups. Individual FG supplementation in HF-feeding conditions also decreased HDL compared to the STD diet. In addition, the FG group presented a higher LDL concentration than the ω-3 group, whereas the combination of both nutrients attenuated the increase in the concentration of plasma LDL induced by FG. Consistent with these results, the FG group showed the highest LDL/HDL ratio value among the groups, and the combination of FG with ω-3 PUFA significantly counteracted this increase. No significant differences were found in plasma TG concentrations among the groups (Table 1).

Isolated ω-3 supplementation attenuated lipid accumulation in the liver under HF-feeding conditions, showing similar TC and even decreased TG concentrations to those seen in the STD group. Conversely, the other groups showed higher TC concentrations than the STD group (Table 1).

No significant differences were observed in the ratio of AST to ALT in plasma among the groups. Interestingly, both transaminase activities tended to decrease in all the rats fed an HF diet compared to those fed a STD diet. The HF group even showed a significantly lower ALT activity than the STD group. The FG&ω-3 combination increased the ALT activity compared to both the FG and the HF groups, with values close to those observed in the STD group (Table 1).

The HF diet did not modify the plasma IL-6 concentration. Interestingly, the FG&ω-3 group showed lower IL-6 concentrations than the STD group (Table 1).

### 3.3. Oxidative Stress Biomarkers in Blood

No significant differences were found in either the non-enzymatic antioxidant capacity or glutathione status in plasma among the groups. However, the HF diet increased plasma lipid peroxidation and albumin carbonylation compared to the STD diet. The combined supplementation with FG and ω-3 PUFA significantly attenuated the increase in the conjugated dienes and tended to decrease albumin carbonylation, mainly modulated by ω-3 PUFA (Table 2).

The HF diet did not modify either antioxidant enzyme activities or glutathione redox state in erythrocytes compared to the STD diet. Nevertheless, some endogenous antioxidant defenses were decreased in the supplemented groups compared to the non-supplemented groups by the end of the study. Although FG and ω-3 supplementations, either individually or combined, decreased the activities of the enzymes of the glutathione system (GPx and GR), the FG&ω-3 group showed a similar GSH content to those observed in the STD and HF groups. Moreover, a lower GSSG content was recorded in the FG&ω-3 group than in the other groups, and this group had the most beneficial glutathione redox state (Table 2).

### 3.4. Oxidative Stress Biomarkers in Tissues

No significant differences were found in antioxidant enzyme activities in adipose tissue between rats fed HF and STD diets. However, the ω-3 group exhibited a higher GSH content than the FG and both non-supplemented groups and had the lowest GSSG/GSH ratio value among the groups. Its combination with FG attenuated the increase in the GSSG/GSH ratio value induced by FG compared to the HF diet without supplementation (Table 3).

In the liver, the FG&ω-3 and HF groups presented a lower SOD activity than the STD group. However, both groups supplemented with ω-3 PUFA, either individually or in combination with FG, had a decreased GSSG content compared to the STD group. The HF diet increased lipid peroxidation and protein carbonylation compared to the STD diet. However, the combined supplementation with FG and ω-3 PUFA attenuated the increase in total protein carbonylation, mainly modulated by ω-3 PUFA (Table 3).

## 4. Discussion

We previously showed that the combined supplementation with FG and ω-3 PUFA attenuates the increase in perigonadal adipose tissue content and the onset of insulin resistance induced by an HF diet [23]. The combination of ω-3 PUFA with another bioactive nutrient may be a useful nutritional strategy for the prevention of obesity and insulin resistance by enhancing anti-inflammatory and antioxidant status [22]. Thus, the present study aimed to examine, for the first time, the combined effects of FG and ω-3 PUFA on dyslipidemia, transaminases, interleukin-6, and oxidative in pre-obese rats.

We observed that the HF diet decreased the TC as well as HDL concentrations in plasma and promoted a slight TC accumulation in the liver compared to the STD diet. Nevertheless, the plasma and liver TG concentrations were unchanged after 21 weeks under HF feeding. This may be because the cholesterol content of the HF diet is too low for the induction of plasma hypercholesterolemia (0.05% of feed), as previously suggested [33]. In addition, the fatty acid overload leads to adaptive responses such as increased fatty acid desaturation and β-oxidation [10,34,35], which may explain why the plasma and liver TG concentrations remained unchanged.

Although none of the supplements counteracted the decrease in plasma HDL induced by the HF diet, the groups supplemented with ω-3 PUFA, either individually or combined with FG, showed lower LDL/HDL values than those found in the FG group. The supplementation with the combination of FG and ω-3 PUFA did not mitigate the slight TC accumulation in the liver induced by the HF diet. On the contrary, individual ω-3 PUFA supplementation prevented TC accumulation, with the rats in that group registering a similar TC and even a lower TG content than the rats fed an STD diet. Other authors have shown that the ω-3 PUFA supplementation exerts plasma hypocholesterolemic effects under HF-feeding conditions [34,36,37]. Thus, ω-3 PUFA supplementation may decrease lipid accumulation in the liver by increasing the ω-3 PUFA content [22,38], which may stimulate β-oxidation [39,40] as well as cholesterol uptake from plasma, bile acid synthesis, and its extraction [41]. In addition, ω-3 PUFA supplementation may decrease cholesterol and TG synthesis [40].

Even though the HF diet induced a slight TC accumulation in the liver, we found no differences in the AST/ALT ratio in plasma among the groups. Increased circulating transaminases are indicators of liver injury and liver metabolic functioning [42]. Interestingly, the HF group exhibited a lower plasma ALT concentration than the STD group. This difference may be associated with decreased muscle mass [43], which is a common characteristic of obesity [44]. Thus, the combination of FG and ω-3 PUFA counteracted the reduction in ALT in plasma with a concomitant decrease in the perigonadal adipose tissue content. Moreover, other studies have shown in rodents that ω-3 PUFA may improve skeletal muscle metabolic function by activating the AMPK/PGC-1α signaling pathway [45] and may decrease proteolysis in the liver by the down-regulation of l-serine dehydratase/l-threonine deaminase, dipeptidyl peptidase 1 light chain, and proteasome subunit beta type-8 [40]. The ω-3 PUFA supplementation may also increase protein synthesis in older adults by activating the mTOR-p70s6k signaling pathway [46].

In the present study, the HF diet did not modify the plasma IL-6 concentration, as observed in other studies in rodents [47]. Low-grade chronic inflammation is a characteristic of obesity [48]. Furthermore, inflammation, rather than hypercholesterolemia, has been identified as a key player in the onset of chronic diseases [21,49]. In fact, other authors have shown in rodents that an HF diet increases the plasma IL-6 concentration [9,38]. Nevertheless, it is possible to observe increased circulating inflammatory biomarkers once obesity and insulin resistance are fully established [9]. Interestingly, the combination of FG and ω-3 PUFA decreased IL-6 compared to the STD group. Previous studies have shown that FG prevents the dysbiosis of gut microbiota induced by excessive fat consumption, maintaining intestinal barrier integrity [13,23]. Furthermore, ω-3 PUFA and its oxidized metabolites exert anti-inflammatory effects [22,50,51,52] by binding to G-protein-coupled receptor 120 and increasing peroxisome proliferator-activated receptor γ, inhibiting the nuclear transcription factor-kappa β pro-inflammatory signaling pathway [37,51,53,54]. Nevertheless, in the present study we observed no significant effects of individual supplementations on plasma IL-6. The decrease in plasma IL-6 in rats receiving the combined supplementation compared to those fed the STD diet might be explained by the distinct fatty acid composition of the dietary backgrounds (STD or HF) and the use of fish oil instead of soybean oil. As we previously reported in the same cohort of rats [32], the STD diet increases the ω-6 PUFA content in the liver compared to the HF diet. The soybean oil is rich in linoleic acid (LA, 18:2 ω-6) (Table S3), which is a precursor of arachidonic acid (ARA, 20:4 ω-6) [55]. Non-esterified ARA can be oxidized producing a large number of lipid mediators related to the induction of pro-inflammatory pathways [55]. Hence, when fish oil is used instead of soybean oil, FG may participate in modulating inflammatory parameters such as plasma IL-6, which is mainly derived from adipose tissue.

We observed that the HF diet induced early stages of insulin resistance, accompanied by increased lipid peroxidation and protein carbonylation in plasma and the liver compared to the STD diet. In agreement with our findings, Ciapaite et al. [35] have shown that an HF diet increases the content of thiobarbituric acid reactive substances and total protein carbonyls in the liver. A previous study found that an HF diet induces an early antioxidant response against the excessive production of reactive oxygen species (ROS) in both adipose tissue and the liver in mice before an advanced insulin-resistant state [10]. It is important to note that, whereas physiological ROS production may enhance insulin sensitivity in the onset of insulin resistance [56], long-term ROS overproduction leads to oxidative stress and inflammation, promoting irreversible oxidative damage and subsequent metabolic alterations [7,11,57].

The combined supplementation with FG and ω-3 PUFA attenuated the lipid peroxidation in plasma and total protein carbonylation in the liver induced by the HF diet. Furthermore, the FG&ω-3 group showed the lowest GSSG content in erythrocytes, accompanied by decreased GPx and GR activities. In fact, the GSSG content in erythrocytes has been suggested as the best oxidative stress biomarker to describe the redox status of tissues [58]. The combined supplementation also decreased the SOD activity as well as the GSSG content in the liver compared to the STD diet, which could suggest decreased ROS production and enhanced antioxidant status in this group, as observed in a previous study [22]. In the present study, the individual supplementation with ω-3 PUFA significantly decreased oxidative damage under HF-feeding conditions. On the contrary, the individual supplementation with FG did not attenuate high-fat diet-induced oxidative stress. Furthermore, the combination of the two supplements attenuated the redox imbalance (i.e., GSSG/GSH) in the adipose tissue induced by FG. Concretely, GSH is a low-molecular weight thiol containing glutamic acid, cysteine, and glycine, ubiquitously present in mammalian tissues but especially in the liver [59]. It is the primary reducing agent both in mitochondria and in the endoplasmic reticulum [60], maintaining a reduced state by either directly or indirectly scavenging ROS [61]. As the GPx and GR activities in the perigonadal adipose tissue were unchanged, the increase in the content of GSH in both ω-3 PUFA-supplemented groups could be provided thought de novo synthesis via the activation of the γ-glutamylcysteine synthetase instead of the recycle process [59]. Taken together, these findings suggest that the beneficial effects of the combination of FG and ω-3 PUFA on oxidative stress were mainly modulated by ω-3 PUFA. It is well-known that dietary ω-3 PUFA and its oxidized metabolites exert antioxidant effects in humans [24], probably by activating the nuclear factor-erythroid 2-related factor 2 signaling pathway [62,63]. Indeed, previous studies have shown that ω-3 PUFA supplementation attenuates the increase in the GSSG/GSH ratio and the content of protein carbonyls in the liver induced by excessive fat intake in rodents [32,38], which is accompanied by enhanced insulin sensitivity [38]. Furthermore, Selenscig et al. [37] have shown that replacing corn oil as a dietary fat source with fish oil improves the epididymal adipose tissue function in rats fed a diet rich in sucrose by increasing antioxidant defenses and reducing ROS production. As far as FG is concerned, other authors have reported that FG attenuates oxidative stress induced by high glucose contents in human umbilical vein endothelial cells by activating the AMPK/SIRT1/PGC-1α pathway [64]. Nevertheless, no previous studies have assessed the effects of FG on antioxidant status in vivo under an HF diet.

## 5. Conclusions

The combined supplementation with FG and ω-3 PUFA partially attenuated oxidative damage to lipids and proteins induced by the HF diet. Furthermore, the combination decreased antioxidant enzyme activities and enhanced glutathione status. The combination did not attenuate the slight accumulation of cholesterol in the liver induced by the HF diet but normalized the plasma alanine aminotransferase activity. The beneficial effects of the combination of FG and ω-3 PUFA on oxidative stress in pre-obese rats were mainly modulated by ω-3 PUFA. Therefore, the increase in the dietary intake of ω-3 PUFA from fish, either individually or in combination with FG from buckwheat, may be a useful nutritional strategy against oxidative stress attenuating the onset of insulin resistance.

## Figures and Tables

**Table 1:** Lipid profile, transaminases and interleukin-6 after 21 weeks of dietary intervention.

	STD	HF	FG	ω-3	FG&ω-3
Plasma lipid profile					
TG (mmol/L)	0.7 ± 0.2	0.5 ± 0.3	0.6 ± 0.1	0.6 ± 0.2	0.4 ± 0.1
TC (mmol/L)	3.6 ± 0.4	2.9 ± 0.5 ^a^	3.1 ± 0.5	2.5 ± 0.4 ^a^	2.6 ± 0.3 ^a^
HDL (mmol/L)	1.15 ± 0.12	0.94 ± 0.16 ^a^	0.94 ± 0.07 ^a^	0.85 ± 0.12 ^a^	0.93 ± 0.07 ^a^
LDL (mmol/L)	0.43 ± 0.11	0.39 ± 0.09	0.47 ± 0.11	0.32 ± 0.07 ^c^	0.36 ± 0.03
LDL/HDL ratio	0.37 ± 0.08	0.42 ± 0.06	0.50 ± 0.11 ^a^	0.38 ± 0.07 ^c^	0.37 ± 0.04 ^c^
Liver lipid profile					
TG (µmol/g tissue)	17.6 ± 1.0	16.6 ± 2.6	14.6 ± 1.3	14.1 ± 2.3 ^a^	16.7 ± 2.5
TC (µmol/g tissue)	5.9 ± 0.7	7.8 ± 1.8 ^a^	7.8 ± 0.8 ^a^	6.4 ± 1.0	7.6 ± 1.0 ^a^
Transaminases and IL-6					
Plasma AST (U/L)	70.2 ± 21.9	57.1 ± 10.7	49.2 ± 8.4	53.6 ± 10.1	61.0 ± 14.4
Plasma ALT (U/L)	27.1 ± 8.2	19.0 ± 3.9 ^a^	20.2 ± 4.7	22.8 ± 4.2	24.0 ± 3.6 ^b,c^
Plasma AST/ALT ratio	2.64 ± 0.52	3.14 ± 0.92	2.49 ± 0.45	2.37 ± 0.29	2.53 ± 0.38
Plasma IL-6 (pg/mL)	522 ± 307	283 ± 167	452 ± 259	349 ± 184	154 ± 72 ^a^

Values are expressed as mean ± standard deviation, nine rats per group. Abbreviations: STD, Standard group; HF, High-Fat group; FG, Fagomine group; ω-3, ω-3 PUFA group; FG&ω-3, Fagomine and ω-3 PUFA group; TG, Triacylglycerol; TC, Total Cholesterol; HDL, High-Density Lipoprotein cholesterol; LDL, Low-Density Lipoprotein cholesterol; AST, Aspartate aminotransferase; ALT, Alanine aminotransferase; IL-6, Interleukin-6. *p*-value was calculated by the one-way analysis of variance or the non-parametric Kruskal-Wallis test followed by Scheffé post-hoc test or Mann–Whitney U test, respectively. ^a^, vs. STD group; ^b^, vs. HF group; ^c^, vs. FG group.

**Table 2:** Oxidative stress biomarkers in blood after 21 weeks of dietary intervention.

	STD	HF	FG	ω-3	FG&ω-3
Plasma					
ORAC (µmol trolox Eq/mL)	18.3 ± 3.8	17.4 ± 4.7	18.4 ± 5.7	21.2 ± 6.1	21.1 ± 7.6
FRAP (µmol trolox Eq/mL)	0.16 ± 0.03	0.14 ± 0.03	0.12 ± 0.02	0.12 ± 0.01	0.13 ± 0.03
GSH (nmol/mL)	9.9 ± 1.4	11.2 ± 3.8	11.4 ± 6.2	10.4 ± 1.9	9.9 ± 3.2
GSSG (nmol/mL)	32.4 ± 2.3	30.5 ± 3.3	29.6 ± 4.9	29.7 ± 4.9	29.3 ± 1.9
GSSG/GSH ratio	3.24 ± 0.57	2.96 ± 0.85	3.19 ± 1.57	2.88 ± 0.41	3.22 ± 1.00
Conjugated dienes (mmol hydroperoxides/kg lipid)	0.13 ± 0.03	0.21 ± 0.06 ^a^	0.17 ± 0.03 ^a^	0.14 ± 0.01 ^b^	0.14 ± 0.02
Albumin carbonylation index	0.17 ± 0.04	0.42 ± 0.14 ^a^	0.36 ± 0.08^a^	0.26 ± 0.05 ^a,b,c^	0.31 ± 0.06 ^a^
Erythrocytes					
SOD (U/g Hb)	1155 ± 410	1022 ± 279	1156 ± 245	1250 ± 367	968 ± 289
CAT (mmol/g Hb)	77.5 ± 11.4	86.6 ± 22.3	71.4 ± 12.8	64.1 ± 6.9	70.1 ± 18.6
GPx (U/g Hb)	85.4 ± 14.7	83.4 ± 13.1	76.7 ± 13.2	71.3 ± 6.8 ^a,b^	61.3 ± 14.4 ^a,b,c^
GR (U/g Hb)	0.31 ± 0.06	0.24 ± 0.04	0.21 ± 0.07 ^a^	0.18 ± 0.03 ^a^	0.19 ± 0.05 ^a^
GSH (µmol/g Hb)	1.51 ± 0.49	1.16 ± 1.06	0.69 ± 0.22 ^a^	0.78 ± 0.57 ^a^	1.69 ± 0.53 ^c,d^
GSSG (µmol/g Hb)	0.85 ± 0.26	0.77 ± 0.28	0.82 ± 0.30	0.93 ± 0.44	0.43 ± 0.08 ^a,b,c,d^
GSSG/GSH ratio	0.60 ± 0.25	1.29 ± 0.99	1.21 ± 0.34	1.56 ± 0.87	0.29 ± 0.11 ^d^

Values are expressed as mean ± standard deviation, nine rats per group. Abbreviations: STD, Standard group; HF, High-Fat group; FG, Fagomine group; ω-3, ω-3 PUFA group; FG&ω-3, Fagomine and ω-3 PUFA group; ORAC, Oxygen Radical Absorbance Capacity; FRAP, Ferric Reducing Ability of Plasma; SOD, Superoxide Dismutase; Hb, Hemoglobin; CAT, Catalase; GPx, Glutathione Peroxidase; GR, Glutathione Reductase; GSH, Reduced Glutathione; GSSG, Oxidized Glutathione. *p*-value was calculated by the one-way analysis of variance or the non-parametric Kruskal–Wallis test followed by Scheffé post-hoc test or Mann-Whitney U test, respectively. ^a^, vs. STD group; ^b^, vs. HF group; ^c^, vs. FG group; ^d^, vs. ω-3 group.

**Table 3:** Oxidative stress biomarkers in tissues after 21 weeks of dietary intervention.

	STD	HF	FG	ω-3	FG&ω-3
Perigonadal white adipose tissue					
SOD (U/g tissue)	83.9 ± 13.1	75.5 ± 7.0	80.8 ± 28.8	63.0 ± 18.2	74.7 ± 24.4
CAT (mmol/g tissue)	0.11 ± 0.01	0.07 ± 0.04	0.10 ± 0.04	0.10 ± 0.03	0.08 ± 0.06
GPx (U/g tissue)	0.25 ± 0.23	0.07 ± 0.06	0.15 ± 0.20	0.05 ± 0.04	0.05 ± 0.05
GR (U/g tissue)	0.15 ± 0.09	0.10 ± 0.08	0.14 ± 0.05	0.14 ± 0.07	0.12 ± 0.07
GSH (nmol/g tissue)	6.79 ± 1.23	6.71 ± 2.79	5.75 ± 2.84	10.92 ± 0.93 ^a,b,c^	8.24 ± 3.76
GSSG (nmol/g tissue)	28.3 ± 6.7	24.2 ± 6.4	26.2 ± 4.5	27.3 ± 4.3	23.5 ± 5.1
GSSG/GSH ratio	4.32 ± 1.49	3.87 ± 0.86	5.31 ± 1.82 ^b^	2.52 ± 0.54 ^a,b,c^	3.13 ± 0.96 ^c^
Liver					
SOD (U/g tissue)	4523 ± 271	2896 ± 883 ^a^	4122 ± 1556	3714 ± 917	2956 ± 379 ^a^
CAT (mmol/g tissue)	17.7 ± 0.9	15.5 ± 1.4	16.1 ± 1.7	15.9 ± 2.5	17.2 ± 2.4
GPx (U/g tissue)	40.7 ± 6.1	34.0 ± 5.2	37.9 ± 2.3	38.3 ± 8.0	36.3 ± 5.6
GR (U/g tissue)	7.86 ± 1.37	5.46 ± 1.18	6.05 ± 1.49	6.34 ± 1.34	7.84 ± 2.64
GSH (µmol/g tissue)	0.86 ± 0.37	0.47 ± 0.29	0.62 ± 0.31	0.58 ± 0.37	0.57 ± 0.40
GSSG (µmol/g tissue)	1.56 ± 0.26	1.29 ± 0.14	1.22 ± 0.07	1.21 ± 0.26 ^a^	1.08 ± 0.28 ^a^
GSSG/GSH ratio	2.02 ± 0.65	3.46 ± 1.55	2.45 ± 1.15	2.97 ± 2.04	2.90 ± 2.10
Conjugated dienes (mmol hydroperoxides/kg lipid)	15.8 ± 0.5	23.3 ± 1.1 ^a^	23.8 ± 1.3 ^a^	21.7 ± 1.1 ^a^	22.6 ± 1.6 ^a^
Total protein carbonylation index	0.74 ± 0.11	0.94 ± 0.16 ^a^	0.91 ± 0.16 ^a^	0.75 ± 0.10 ^b,c^	0.63 ± 0.08 ^b,c^

Values are expressed as mean ± standard deviation, nine rats per group. Abbreviations: STD, Standard group; HF, High-Fat group; FG, Fagomine group; ω-3, ω-3 PUFA group; FG&ω-3, Fagomine and ω-3 PUFA group; SOD, Superoxide Dismutase; CAT, Catalase; GPx, Glutathione Peroxidase; GR, Glutathione Reductase; GSH, Reduced Glutathione; GSSG, Oxidized Glutathione. *p*-value was calculated by the one-way analysis of variance or the non-parametric Kruskal–Wallis test followed by Scheffé post-hoc test or Mann–Whitney U test, respectively. ^a^, vs. STD group; ^b^, vs. HF group; ^c^, vs. FG group.

## Data Availability

The data presented in this study are available on request from the corresponding author.

## Supplementary Materials

The following are available online at https://www.mdpi.com/2304-8158/10/2/332/s1: Table S1: Diet composition; Table S2: Fatty acid composition (mol %) of soybean and fish oils; Figure S1: (A) Energy intake and (B) body weight throughout the study; Table S3: Feed intake, biometric data and insulin resistance biomarkers after 21 weeks of dietary intervention.
