# Supplementary material for: The Effects of the Combination of Buckwheat D-Fagomine and Fish Omega-3 Fatty Acids on Oxidative Stress and Related Risk Factors in Pre-Obese Rats
Source: Foods. 2021 Feb 4;10(2):332. doi: 10.3390/foods10020332 (PMC7913974; doi:10.3390/foods10020332)
Supplement: Supplementary file 1 [file foods-10-00332-s001.pdf]

Electronic Supplementary Information

The Effects of the Combination of Buckwheat D-Fagomine and Fish Omega-3 Fatty Acids on Oxidative Stress and Related Risk Factors in Pre-Obese Rats

Bernat Miralles-Pérez [1], Maria Rosa Nogués [1,*], Vanessa Sánchez-Martos [1], Núria Taltavull [1], Lucía Méndez [2], Isabel Medina [2], Sara Ramos-Romero [3,4], Josep L. Torres [3] and Marta Romeu[1]

[1] Universitat Rovira i Virgili, Department of Basic Medical Sciences, Pharmacology Unit, Functional Nutrition, Oxidation and Cardiovascular Disease Research Group (NFOC-SALUT), C/ Sant Llorenç 21, E-43201 Reus, Spain; bernat.miralles@urv.cat (B.M.P.); vanessa.sanchez@urv.cat (V.S.-M.); nuria.taltavull@urv.cat (N.T.); marta.romeu@urv.cat (M.R.)

[2] Institute of Marine Research (IIM-CSIC), C/ Eduardo Cabello 6, E-36208 Vigo, Spain; luciamendez@iim.csic.es (L.M.); medina@iim.csic.es (I.M.)

[3] Institute of Advanced Chemistry of Catalonia (IQAC-CSIC), C/ Jordi Girona 18-26, E-08034 Barcelona, Spain; sara.ramos@iqac.csic.es (S.R.-R.); josepllluis.torres@iqac.csic.es (J.L.T.)

[4] Current address: Department of Cell Biology, Physiology & Immunology, Faculty of Biology, University of Barcelona, Avd/ Diagonal 643, E-08028 Barcelona, Spain

* Correspondence: mariarosa.nogues@urv.cat (M.R.N.); Tel.: +34-977-75-9378

Table S1. Diet composition.

	STD	HF	FG	ω-3	FG&ω-3
**Diet composition**					
Flour (g)	1,000[a]	1,000[b]	1,000[b]	1,000[b]	1,000[b]
Fagomine (g)	-	-	0.96	-	0.96
Oil	0.8 mL SBO/kg b.w.	0.8 mL SBO/kg b.w.	0.8 mL SBO/kg b.w.	0.8 mL FO/kg b.w.	0.8 mL FO/kg b.w.
**Macronutrients (% weight)**					
Protein	14.3	17.0	17.0	17.0	17.0
Fat	4.0	23.0	23.0	23.0	23.0
Carbohydrates	48.0	47.6	47.6	47.6	47.6
**Macronutrients (% caloric value)**					
Protein	20.0	14.7	14.7	14.7	14.7
Fat	13.0	40.6	40.6	40.6	40.6
Carbohydrates	67.0	40.7[c]	40.7[c]	40.7[c]	40.7[c]
Total energy density (kcal/g)[d]	2.9	4.7	4.7	4.7	4.7

Abbreviations: STD, Standard Group; HF, High-Fat Group; FG, Fagomine Group; ω-3, ω-3 PUFA Group; FG&ω-3, Fagomine and ω-3 PUFA Group; SBO, Soybean Oil; FO, Fish Oil; b.w., Body Weight. [a]Teklad Global 14% Protein Rodent Maintenance Diet (Envigo, IN, USA), [b]TD.08811 45% kcal Fat Diet (Envigo, IN, USA), [c]Carbohydrate (available) is calculated by subtracting neutral detergent fiber from total carbohydrates, [d]Energy density was calculated as estimates of metabolizable energy based on the Atwater conversion factors, assigning 4 kcal/g of protein, 9 kcal/g of fat, and 4 kcal/g of available carbohydrate.

Table S2. Fatty acid composition (mol %) of soybean and fish oils.

	**SBO**	**FO**
14:00	0.96 ± 0.02	4.37 ± 0.05
15:00	0.15 ± 0.01	0.29 ± 0.02
16:00	17.78 ± 0.10	10.15 ± 0.16
16:1 ω-7	0.90 ± 0.03	4.99 ± 0.04
17:00	0.21 ± 0.01	0.45 ± 0.004
18:00	2.07 ± 0.01	2.94 ± 0.03
18:1 ω-9	18.75 ± 0.03	6.41 ± 0.06
18:1 ω-7	1.52 ± 0.02	1.91 ± 0.03
18:2 ω-6	47.55 ± 0.01	0.65 ± 0.01
20:00	n.d	0.32 ± 0.01
18:3 ω-3	4.00 ± 0.04	0.36 ± 0.01
20:1 ω-9	1.43 ± 0.09	0.98 ± 0.03
18:4 ω-3	0.15 ± 0.004	1.51 ± 0.02
20:2 ω-6	0.20 ± 0.05	0.21 ± 0.003
20:3 ω-6	n.d	0.22 ± 0.01
20:4 ω-6	0.40 ± 0.02	1.68 ± 0.04
22:1 ω-11	1.08 ± 0.01	1.14 ± 0.01
22:1 ω-9	0.25 ± 0.02	0.28 ± 0.03
20:4 ω-3	0.20 ± 0.03	1.02 ± 0.02
20:5 ω-3	0.70 ± 0.02	25.09 ± 0.10
24:1 ω-9	0.28 ± 0.05	0.38 ± 0.003
22:5 ω-3	0.26 ± 0.01	4.30 ± 0.05
22:6 ω-3	1.15 ± 0.03	25.70 ± 0.21
ω-3	6.47 ± 0.14	58.84 ± 0.16
SFA	21.17 ± 0.10	18.52 ± 0.22
MUFA	24.21 ± 0.11	17.22 ± 0.12
PUFA	54.62 ± 0.03	64.26 ± 0.33
EPA+DHA	1.85 ± 0.06	50.79 ± 0.31

Abbreviations: SBO, Soybean Oil; FO, Fish Oil; SFA; n.d., non-detected; SFA, Saturated Fatty acids; MUFA, Monounsaturated Fatty Acids; PUFA, Polyunsaturated Fatty Acids; EPA, Eicosapentaenoic; DHA, Docosahexaenoic.

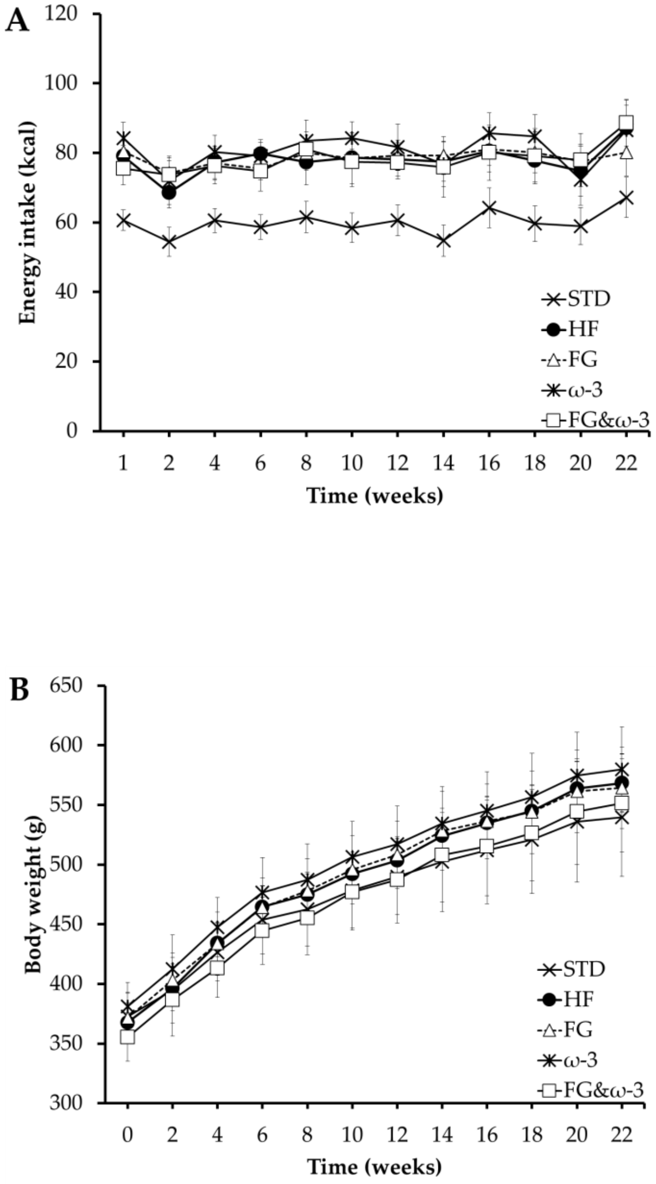

Figure S1. (**A**) Energy intake and (**B**) body weight throughout the study. Values are expressed as mean ± standard deviation, nine rats per group. Abbreviations: STD, Standard group; HF, High-Fat group; FG, Fagomine group; ω-3, ω-3 PUFA group; FG&ω-3, Fagomine and ω-3 PUFA group. Groups fed a HF diet either with supplement or without supplement showed significantly higher energy intake than the STD group (p-value < 0.05). Nevertheless, no significant differences were observed in body weight among the groups.

Table S3. Feed intake, biometric data and insulin resistance biomarkers after 21 weeks of dietary intervention*

	STD	HF	FG	ω-3	FG&ω-3
**Intake data**					
Feed intake (g/day)	20.3 ± 1.6	16.3 ± 0.9[a]	16.4 ± 1.2[a]	16.8 ± 1.0[a]	15.9 ± 1.2[a]
Energetic intake (kcal/day)	58.9 ± 4.6	76.5 ± 4.3[a]	77.2 ± 5.7[a]	79.0 ± 4.7[a]	74.8 ± 5.7[a]
**Biometric data**					
Initial body weight (g)	373 ± 20	368 ± 17	370 ± 22	381 ± 20	355 ± 20
Final body weight (g)	540 ± 49	568 ± 24	564 ± 34	580 ± 36	552 ± 41
Body weight gain (g)	167 ± 36	201 ± 29	194 ± 21	199 ± 30	196 ± 24
Perigonadal adipose tissue (g)	8.1 ± 1.7	13.1 ± 3.9[a]	10.8 ± 1.3	13.3 ± 4.4[a]	9.9 ± 2.1
Adiposity index (%)	1.5 ± 0.3	2.3 ± 0.7[a]	1.9 ± 0.3	2.3 ± 0.6[a]	1.8 ± 0.3
Liver (g)	14.0 ± 1.7	14.0 ± 1.1	14.6 ± 1.0	14.1 ± 1.0	14.6 ± 2.3
Hepatosomatic index (%)	2.6 ± 0.1	2.4 ± 0.2	2.6 ± 0.1	2.5 ± 0.1	2.6 ± 0.3
**Insulin resistance biomarkers**					
OGTT[#] (AUC, mg/mL per 120 min)	10,350 ± 739	10,335 ± 282	10,623 ± 475	10,864 ± 625	10,338 ± 604
Glucose (mmol/L)	3.5 ± 0.4	3.9 ± 0.3[a]	3.9 ± 0.3[a]	3.9 ± 0.3[a]	3.8 ± 0.2
Insulin (mU/L)	16.2 ± 9.4	52.1 ± 23.9[a]	39.3 ± 19.5	42.1 ± 20.9	40.9 ± 16.5

Values are expressed as mean ± standard deviation, nine rats per group. Abbreviations: STD, Standard group; HF, High-Fat group; FG, Fagomine group; ω-3, ω-3 PUFA group; FG&ω-3, Fagomine and ω-3 PUFA group; OGTT, Oral Glucose Tolerance Test; AUC, Area Under the Curve. *These parameters have partially been published in a previous report [1]. [#]OGTT was performed during week 18.

References

1. Hereu, M.; Ramos-Romero, S.; Busquets, C.; Atienza, L.; Amézqueta, S.; Miralles-Pérez, B.; Nogués, M.R.; Méndez, L.; Medina, I.; Torres, J.L. Effects of combined d-fagomine and omega-3 PUFAs on gut microbiota subpopulations and diabetes risk factors in rats fed a high-fat diet. *Sci. Rep.* **2019**, *9*, 1–12, doi:10.1038/s41598-019-52678-5.
